# Safe Milk

**Published:** 1920-04-03

**Authors:** 

**Affiliations:** Chairman of the Council, Grade A Milk Producers' Association. 11 St. James' Square, S.W. 1.


### THE EDITOR'S LETTER-BOX.

(Correspondence on all subjects is invited, but we cannot in any way be responsible for the opinions expressed by our correspondents, who must give their name and address as a guarantee of good faith, but not necessarily for publication. Correspondents are reminded that brevity of style and conciseness of statement greatly facilitate early insertion.)

### SAFE MILK.

### VISCOUNT ELVEDEN ON THE GOVERNMENT'S EFFORTS.

*To the Editor of* The Hospital.

Sir,—I have read with interest your article in this week's issue of The Hospital entitled "Safe Milk." In this connection I am disappointed to see no allusion made to the efforts of the present Ministries of Health and Food in this direction.

Two qualities of milk are now on the market, sold under licence from the above-named Ministries as Grade A (Certified) and Grade A milk. As regards both these grades, frequent inspection renders the possibility of "trouble or expense being spared" very slight on a farm licensed to sell graded milk, even in the remote rural districts. Inspectors visit the farms constantly, however remote they may be and irrespective of whether the product is retailed in large or small towns. Grade A (Certified) milk must, under the regulations, be bottled on the farm where it is produced, and must not contain at any time before reaching the consumer more than 30,000 bacteria per cubic centimetre, or bacillus coli in one-tenth cubic centimetre. Grade A milk must be delivered by dealers in the vessels in which they receive it or in bottles filled on the dealers' premises, which are regularly inspected.

Both Grade A (Certified) and Grade A milk must be produced by herds that contain no animal that reacts to the tuberculin test, on farms that obtain for dairy equipment and methods not less than 300 out of a possible maximum of 500 marks on the Government inspection card. The number of farms at present supplying this milk is small but growing, and it only remains for the public and the medical profession to demand this milk to ensure a plentiful supply.—Yours faithfully,

Elveden.

Chairman of the Council, Grade A Milk Producers' Association.

11 St. James' Square, S.W. 1.

March 26, 1920.

[We are glad that attention is drawn to the beginnings of what should prove to be a most valuable form of Governmental action in the clean milk campaign. Practical co-operation and understanding between the Ministries, the producers, and the public are urgently needed, and particularly is this so in the smaller townships of provincial England.]

### SANITARY BLOCKS OF HOSPITALS.

*To the Editor of* The Hospital.

